# Farmed Gilthead Sea Bream (*Sparus aurata*) by-Products Valorization: Viscera Oil ω-3 Enrichment by Short-Path Distillation and In Vitro Bioactivity Evaluation

**DOI:** 10.3390/md19030160

**Published:** 2021-03-18

**Authors:** Concetta Maria Messina, Rosaria Arena, Simona Manuguerra, Giuseppe Renda, Vincenzo Alessandro Laudicella, Giovanna Ficano, Gioacchino Fazio, Laura La Barbera, Andrea Santulli

**Affiliations:** 1 Laboratorio di Biochimica Marina ed Ecotossicologia, Dipartimento di Scienze della Terra e del Mare DiSTeM, Università degli Studi di Palermo, Via G. Barlotta 4, 91100 Trapani, Italy; concetta.messina@unipa.it (C.M.M.); rosaria.arena@unipa.it (R.A.); simona.manuguerra@unipa.it (S.M.); giuseppe.renda02@unipa.it (G.R.); giovanna.ficano@unipa.it (G.F.); 2 Istituto di Biologia Marina, Consorzio Universitario della Provincia di Trapani, Via G. Barlotta 4, 91100 Trapani, Italy; alessandro.laudicella@gmail.com (V.A.L.); labarbera@consunitp.it (L.L.B.); 3 Dipartimento di Science Economiche, Aziendali e Statistiche, DSEAS, Università degli Studi di Palermo, Viale delle Scienze, Edificio 13, 90100 Palermo, Italy; gioacchino.fazio@unipa.it

**Keywords:** aquaculture, by-products, omega-3 fatty acids, fish oils, nutraceutics

## Abstract

This study shows a pilot scale protocol aimed to obtain an omega 3-enriched oil after the processing of farmed gilthead sea bream viscera (SBV); this was oil was tested in vitro for bioactivity, attesting to the possibility to turn waste into profit The quality of the oil, in terms of requirements for animal and human consumption, was assessed by determining some chemical parameters, such as peroxide value (PV), thiobarbituric acid reactive substances (TBARS), ρ-anisidine (ρ-AV) content, total oxidation value (TOTOX), and phospholipids and free fatty acid (%), both in crude viscera oil (CVO) and refined viscera oil (RVO). Among the extraction conditions, the higher CVO yields were obtained at 60 °C for 10 min (57.89%) and at 80 °C for 10 min (67.5%), and the resulting oxidation levels were low when utilizing both extraction conditions. RVO, obtained from CVO extracted at 60 °C, showed the highest quality on the basis of the assessed parameters. The ethyl esters of the total fatty acid (TFA) contents extracted from RVO were enriched in the ω-3 polyunsaturated fatty acid fraction (PUFAE) up to almost 56% via short path distillation (SPD). Antioxidant activities and adipogenic properties were tested in vitro. PUFAE protected 3T3 L1 cells from oxidative stress and exerted an anti-adipogenic effect in *Dicentrarchus labrax* pre-adipocytes, attesting to the beneficial properties for both farmed fish and human health. These results could stimulate the adoption of solutions aimed to recover and utilize aquaculture by-products at a higher scale, turning “waste into profit” and indicating a strategy to reach more sustainable business models in aquaculture resource utilization according to the principles of the circular economy.

## 1. Introduction

The increasing pressure on natural resources has resulted in an urgent need to optimize the destiny of the by-products of the main food supply chains.

The reduction of food loss and waste, as well as their valorization, is crucial to achieve the goal of “zero waste”. Considering that environmental sustainability is closely linked to economic sustainability, scientific and technical knowledge is essential to outline the road map of more sustainable business models and to optimize the efficiency of aquatic resource utilization [1,2,3], according to the 2030 agenda. In view to target the 14th Sustainable Development Goal (SDG) of the United Nations Development Programme (UNDP) (devoted to the proper utilization of the “resources below the water”) and considering the pivotal role of aquaculture in satisfying the global demand for fish products, the contribution of this industry to the production of by-products must be properly managed at the national and regional levels.

It is well-known that marine oils are an excellent source of ω-3 long chain, polyunsaturated fatty acids (PUFAs), including eicosapentaenoic acid (EPA) and docosahexaenoic acid (DHA). These particular classes of PUFA are common in marine organisms; in particular, PUFAs are synthesized in microalgae and tend to significantly accumulate in fatty fish and in the oil extracted from these organisms and their by-products [1,4,5,6,7,8,9].

In recent years, there has been an exponential growth in the market of the ω-3 PUFAs for human consumption, thanks to numerous studies showing the significant beneficial effects determined by fish oil and ω-3 PUFA-rich functional foods daily consumption in terms of dietetic [6,10] anti-tumor [6,11,12,13,14], and antithrombotic properties [5,15,16].

In addition, the demand for ω-3 PUFA fish oils for animal feeds, in particular for aquaculture, has also been rapidly growing [17,18,19] due to the recognized beneficial effects on growth performance, nutritional value [20,21,22,23], and immune system of reared fish [24].

As the industrial production of fish oil is based on the intensive fishing of fatty fish species belonging to the families such as Scomberesocidae, Gadidae, and Clupeidae, with a consequent depletion of wild fish stocks, it is important to identify new sources for the industrial production of PUFA-rich oils and ω-3 PUFA concentrates [7,25].

In this context, the use of fisheries and aquaculture by-products and wastes as raw material can be an important resource that still contains a large amount of components with high nutritional value, such as ω-3 PUFA [7,16,26,27,28,29,30,31,32].

By-products from processed farmed fish are extremely interesting because they are obtained from a highly controlled processing chain [19], with a high organoleptic quality [21] and a high content of oils and fats that—if properly stored, processed, and enriched—can provide a high amount of ω-3 PUFA for direct human consumption [33,34] with significant beneficial effects [3,6,10,11,16]. In particular, in the wide range of beneficial effects, several studies have suggested that ω-3 PUFA also has antioxidant, anti-inflammatory, and anti-adipogenic effects. These effects, with important nutritional and nutraceutical implications, have been demonstrated in vitro on different cell lines [35,36,37,38].

The utilization of by-products from fisheries and aquaculture, according to the principles of the circular economy, will turn “waste into profit”, indicating more sustainable business models and optimizing the efficiency of aquatic resource utilization [1]. Companies, in fact, will have a direct economic return from the commercialization of bio-products and an indirect return from the reduction waste to be sent to landfills [25,39,40]. This last aspect will also allow for a positive environmental impact by reducing the pressure on the environment of these productive activities [8].

About 30% of the total marine aquaculture production in Sicily (around 2.000 t/year) is minimally processed (gilled, gutted, and fillet) and marketed at local markets or in large-scale distribution. We estimate that, on the regional scale, reared seabass and sea bream processing guarantees a production of 36/40 t/year of viscera, with this component being about 6/7% of the total by-product.

In the main Sicilian fish farm, wastes from processed fish are automatically collected during the production cycle, stored, and sent to landfills. In agreement with this farm, as part of an industrial research project, we developed a pilot process for the recovery and valorization of this waste with the aim to turn “waste into profit” and contribute to increasing the economic and environmental sustainability of aquaculture as a paradigm of the circular economy.

The aim of this research was to develop and optimize methods for the separation of crude viscera oil (CVO) taken from reared sea bream (*Sparus aurata*) viscera (SBV) and its refinement to obtain refined viscera oil (RVO) by short path distillation (SPD). After transesterification to produce total fatty acid (TFA) ethyl esters from RVO, SPD was employed to enrich the ω-3 PUFA fraction (PUFAE) and separate the exhausted fatty acid ethyl esters fraction (EFA). On refined oil and separated fractions, bioactive properties were evaluated in vitro to investigate the potential antioxidant effects in the 3T3-L1 cell line and anti-adipogenic effects in primary pre-adipocytes of *Dicentrarchus labrax.*

## 2. Results and Discussion

### 2.1. Proximate Composition of Sea Bream Viscera (SBV) By-Product

The proximate composition of SBV (Table 1) showed a high total lipid content (51.79 ± 12.92%) and moisture, protein, and ash values of 40.81 ± 4.86%, 5.67 ± 0.02%, and 1.43 ± 0.55%, respectively (Table 1).

The total lipid content was higher than the data reported in the literature by Pateiro et al. [8] (34.11% in the guts and 25.76% in the liver of reared sea bream), Rincón Cervera et al. [33] (34% in the viscera of sea bream from semi-extensive farming), and Sinanoglou et al. [41] (29.92 ± 3.5% under organic and 42.61 ± 5.29% under conventional production systems). Kandyliari et al. [42] showed a total lipid content equal to 43.19% (large size) and 55.12% (small size) in the intestine of sea bream reared in a pilot-scale cage farm.

These diverse data confirm that lipid content in fish tissues is significant influenced by rearing conditions. The reared sea bream, also due to the considerable availability of artificial food, usually shows a greater accumulation of fat when compared to wild specimens, and, as expected, the fatty acid profile tends to reflect that of the administered diet [8,33,41,43,44].

The fatty acid profile of SBV total lipids is shown in Table 2.

Monounsaturated fatty acids (MUFAs) were the most abundant class of fatty acids (49.13 ± 0.76%), followed by PUFAs (27.38 ± 0.48%) and saturated fatty acids (SFAs) (23.51 ± 0.28%). The predominant fatty acid was oleic acid (18:1n9, 16.11 ± 0.40%) (Table 2). This profile was comparable to results reported by other authors for reared sea bream by-products [8,33,41,42] and was in accordance with the observation that the relative proportion of oleic acid is not strictly affected by the rearing system [41]. For reared species, such as sea bass and sea bream, numerous data in the literature attest to the direct effect of the diets on tissues fatty acid composition and, consequently, on the nutritional, organoleptic and shelf life properties of the products [22,33,41,42,43,44,45,46]. In particular, increasing levels of ω-6 PUFA are known to result from the supplementation of fish feed with vegetable oils, which increases the proportion of dietary C18:2 n-6 [18,41,45,47].

As expected, this parameter is also strictly subjected to quantitative and qualitative variations in relation to life cycle, diet, and other ecological factors [18,22,30,41,45,47]].

Among PUFAs, the predominant fatty acids were EPA and DHA (8.19 ± 0.12% and 11.27 ± 0.30, respectively) (Table 2). Pateiro et al. [8] showed a lower content of total long-chain PUFA but a higher contents of EPA and DHA, while data reported by Rincón Cervera et al. [33] showed a lower content of EPA and a higher content of DHA in sea bass and sea bream by-products [33]. Moreover, the sum of EPA and DHA content, in our study, was higher when compared to that reported by Maschmeyer et al. [30] in sea bass and sea bream by-products, probably as a consequence of the administered diet.

### 2.2. Yield and Quality of Crude Viscera Oil (CVO)

**Figure 1:**
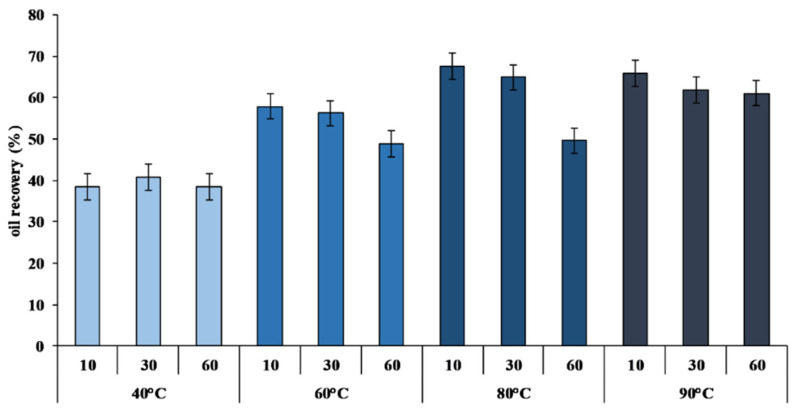
Percentage of crude viscera oil (CVO) recovery calculated on the total lipid content of sea bream viscera (SBV) at different temperatures (40, 60, 80,and 90 °C) and different extraction times (10′, 30′, and 60′) (*n* = 12).

The monitoring of the extraction process was carried out by evaluating the percentage of oil recovery (%) (Figure 1) and the quality parameters, as shown in Table 3.

An increase of CVO yield was observed as the reaction temperature rose (Figure 1). The yield, calculated on the total lipid content of SBV, increased from 38.46 ± 2.43% at 40 °C for 10 min to 67.51 ± 2.60% at 80 °C for 10 min (Figure 1). These results confirmed that, as already reported [48,49,50], higher temperatures are preferential to increase the extraction yield.

Under optimal extraction conditions, without the use of any solvents, the procedure we have described allows us to extract and separate about 6.5 L of oil from 20 kg of viscera in about three hours.

The evaluation of primary and secondary lipid oxidation markers like peroxide value (PV) and thiobarbituric acid reactive substances (TBARS) showed that these compounds were highly influenced by the extraction conditions. It is known that higher temperatures promote lipid oxidation, which leads to a decrease in oil quality [48]. The peroxide content ranged from 5.42 ± 0.13 meq O_2_/kg in CVO extracted at 60 °C for 10 min to a maximum of 22.74 ± 2.27 meq O_2_/kg in CVO extracted at 90 °C for 60 min (Table 3). The maximum PVs were recorded in CVO extracted at 60 min, rather than 10 and 30 min, at each temperature (Table 3) (*p* < 0.05).

The PV in CVO was higher than values registered in the commercial cod liver oil control oil (CO) for human consumption (2.10 ± 0.53 meq O_2_/kg) (Table 3). None of the CVO we extracted from SBV showed a content lower than 5 meq O_2_/kg, so it was necessary to refine it for the human consumption and, in any case, for further applications [51,52].

The increase of oxidation parameters at higher temperatures (Table 3) was comparable to the yield and quality of *Oncorhynchus nerka* [53] and *Clarias gariepinus* [54].

The evaluation of lipid oxidation by TBARS, expressed as the content of malondialdehyde (MDA) in oil (µg/g), showed a comparable trend to the PV content (Table 3). TBARS content, in fact, increased in relation to CVO extraction temperature (Table 3) from 14.15 ± 0.0, at 60 °C for 10 min to a maximum of 33.43 ± 5.63 at 90 °C for 60 min (Table 3). In any case, the TBARS content of CVO was higher than the TBARS content of the reference oil (CO: 5.51 ± 0.81 MDA µg/g) (Table 3). However, the TBARS content of CVO (Table 3) was lower than the values observed by Šimat et al. [25,34] in crude oil extracted from sea bass and sea bream guts.

At higher extraction temperatures, we reported an alteration of CVO color (Table S1). From the CIELab coordinates [55], we observed a variation from yellow (parameter b*) in CVO extracted at 40 and 60 °C to red (parameter a*) in CVO extracted at 80 and 90 °C (Table S1 and Figure S1). Furthermore, the increase of extraction temperature also led to a reduction of CVO lightness (parameter L*) (Table S1). In any case, the color of all our CVO was always different from the color of the reference oil (CO) (*p* < 0.05) (Table S1).

The high temperature extraction-induced lipid oxidation that determined a variation of the extracted oil color [53,56,57,58].

Thus, extracted oil color evaluation could be utilized as quick and cheap oil quality marker.

The variation of the CVO’s main classes of fatty acids, on the basis of extraction temperature, is shown in Table 4.

A significant reduction in PUFA was observed as the temperature increased, with a significantly lower value in the oil extracted at 90° for 60 min (*p* < 0.05). It is known in literature, in fact, that high temperatures lead to the thermal degradation of some polyunsaturated fatty acids [59].

However, at the highest extraction temperature, the EPA and DHA contents were higher than those observed by Šimat et al. [25,34] in crude oil extracted from sea bass and sea bream by-products under similar conditions (95 °C for 12 min).

The evaluation of the yield and quality parameters of the extracted CVO confirmed that the extraction conditions directly affected the oil yield, reaching a maximum value in CVO extracted at 80 °C (Figure 1). In addition, at high temperatures, a higher level of lipid oxidation was observed, as evidenced by the increase in the value of peroxides and TBARS (Table 3), especially at longer reaction times. However, the quality of CVO extracted at 60 °C for 10 min and 80 °C for 10 min was within the guidelines for the evaluation of primary and secondary lipid oxidation markers of unprocessed fish oils that were recently published by the European Food Safety Agency [60]. For this reason, we chose to test the refining process on CVO 60 °C 10 min and CVO extracted at 80 °C 10 min because these the extraction conditions that offered the highest quality combined with a good product yield.

### 2.3. Crude Oil Refining: Effects of the Chemical Processes on Oil Quality

Crude fish oil is not suitable for direct human consumption, and further processing is need [25,52,60,61]. Phospholipids, water, free fatty acids, mono and di-glycerides, pigments, hydrocarbons, sterols, vitamins, pigments, carbohydrates, proteins, and lipid oxidation products may give undesirable flavors and colors [25,52,60,61]. The refining process is necessary to improve the quality of crude fish oil.

With the aim to remove undesirable components, CVO undergoes a refining process to obtain RVO and stabilize it [52,60], as described below.

The effect of the starting CVO quality, extracted at two temperatures (60 and 80 °C) on the different quality parameters of the final RVO [25,52,60,62,63], are shown in Table 5.

Similarly, in the present study, after the refining process of SPD, a decrease in the main parameters related to the lipid components oxidation was observed, in accordance to literature [25,64]. In fact, both primary oxidation components (like PV) and secondary oxidation products (like ρ-anisidine (ρ-AV) and TBARS) showed a significant decrease in RVO produced from CVO that was extracted at 60 and 80 °C (Table 5).

Total oxidation value (TOTOX) also reflected the trend of the previous lipid oxidation markers (Table 5) with a significant decrease in RVO produced from CVO extracted at 60 and 80 °C. These results confirmed the influence of the refining process on oil oxidation status and, in terms of PV and ρ-AV of RVO produced from CVO extracted at 60 °C, had results comparable to those observed by Šimat et al. [25]. The values of TOTOX and TBARS (Table 5) in RVO produced from CVO extracted at 60 °C were lower than those observed by the same authors [25]. The results of this study for the PV, ρ-AV, and TOTOX contents of RVO at 60 °C were comparable to the values observed by Franklin et al. [58] in oil extracted from yellowtail fish waste by supercritical CO_2_ extraction [58].

The contents of phospholipids, which act as emulsifiers and increase oil viscosity [30], have been analyzed to evaluate the effectiveness of the degumming, the first step of the refining process. As already reported [30,65,66], CVO refining determines a reduction of phospholipid content (Table 5) below 10 mg/kg, the optimal suggested value for edible oils [52,60,63,66].

The refining process is also able to remove free fatty acids (FFAs), which are among the most responsible for the characteristic rancid odor as consequence of the oxidation process in the oil [67], thus necessitating the deacidification step in the refining process [66].

Our results showed a significant reduction of FFA in RVO produced from CVO extracted at 60 °C (Table 5), reaching values lower than 3%, which is the threshold recommended for edible oils [52,60].

Colorimetric analyses showed significant differences (*p* < 0.05) among all considered parameters between CVO and RVO (Table S2). These observations were comparable to those reported in the literature for refined fish oils [61,68,69], confirming that the refining process produces more transparent oil, an increase in the luminosity, and a tendency to be yellow [61,68,69].

It is known that during the refining process, the removal of residues and impurities, such as oxidation products, peroxides, phospholipid, metals, soaps, and organic contaminants, leads to a brightening of the oil, as attested to by an increase of the L* parameter [61,68,69].

Kuo et al. [62] observed a different trend with the decrease in the a* value (redness) and increase in the b* value (yellowness) as a result of the removal of some pigments during the cobia liver oil refining process. In fact, the bleaching process could remove some pigments and their secondary products—like aldehydes, ketones, trace metals, and sulfurous compounds—to modify the final color of the refined oil [62].

SPD allows for the use of low temperatures for the deodorization and refining of fish oils, as well as significant decreases of oxidation molecules and free fatty acid contents in treated oil [70]. By utilizing SPD, it is possible to obtain an oil that meet the quality standards for human consumption [52,60,63].

On the basis of the analyzed quality markers, the RVO produced from CVO extracted at 60 °C (Table 5) is the most appropriate oil for a direct human consumption, according to the current guidelines for fish oil for human consumption (PV ≤ 5 meqO_2_/kg; ρ-AV ≤ 20; TOTOX ≤ 26; and AV ≤ 3 mg KOH/g) [52,60,63]. The ω-3 PUFA fraction of RVO produced from CVO extracted at 60 °C was then enriched by SPD.

### 2.4. PUFA Enrichment

RVO transesterification showed an average yield of 65.5 ± 3.5% of TFA.

The obtained TFAs were submitted to SPD to enrich the PUFA content, in the PUFAE fraction, via the elimination of the fraction containing short chain and EFAs.

**Figure 2:**
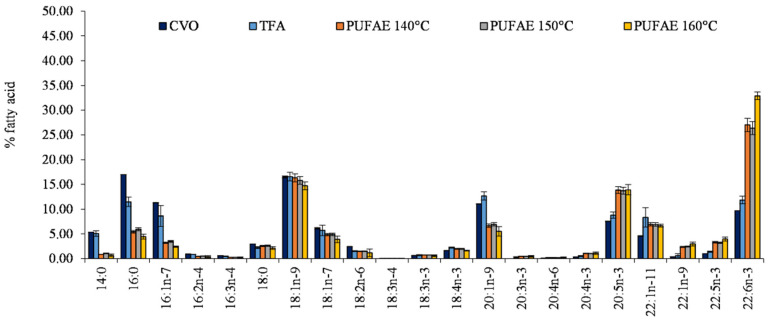
Fatty acid profile (% fatty acids) of total methyl esters obtained by crude viscera oil (CVO), the total ethyl esters of total fatty acid (TFA), and the fraction enriched in PUFA (PUFAE) by short path distillation (SPD) at the three utilized distillation temperatures of 140, 150, and 160 °C. The data are given as an average of the fatty acid profile from 3 distillations.

Fatty acid profiles, determined by gas chromatography, are reported in Figure 2.

Differences among CVO, TFA, and PUFAE fatty acid profiles (Figure 2) confirmed the enrichment effect in PUFA induced by SPD (Figure 2).

After increasing the distillation temperature, an increase of the percentage content of PUFA in PUFAE was observed (Figure 2). In particular, EPA and DHA increased from 8.80% and 11.84% in TFA to 13.85% (EPA) and 27.03% (DHA) in PUFAE separated at 140 °C, as well as up to 13.92% (EPA) and 32.90% (DHA) in PUFAE distillated at 160 °C (Figure 2).

The enrichment of PUFA by SPD is a consequence of the elimination of the short-chain and saturated fatty acids that, under the operation conditions, were distillated in the light phase [70,71].

In fact, after analyzing the percentage fatty acid contents of TFA, PUFAE, and EFA, separated at the distillation temperature of 160 °C that showed the best yield (Table 6), this enrichment of PUFAE and the depletion of EFA was evident.

The observed decrease in short-chain and saturated fatty acid percentage contents in PUFAE was a consequence of their distillation in the light fraction, EFA (Table 6). The concentration of the main short-chain and saturated fatty acid percentage content in TFA—5.07% (14:0), 11.47% (16:0), and 2.24% (18:0)—decreased in PUFAE distillated at 160 °C—0.66% (14:0), 4.45% (16:0), and 2.43% (18:0) (Table 6).

To reduce thermal damage to the long chain PUFA, the highest temperatures used was 160 °C. In fact, although the SPD technique is considered ideal for the separation of highly thermolabile components with minimal thermal degradation [71], operation conditions that include extremely high temperatures that could facilitate the separation of high molecular weight molecules are not recommended [72].

This temperature, in addition, granted the highest PUFA enrichment. PUFA increased in PUFAE (from 28.71% to 56.55%), while in the EFA fraction, it was depleted (30.19%) (Table 6). Short-chain and saturated fatty acid percentage contents showed a significant decrease (*p* < 0.05) in PUFAE separated at 160 °C by SPD respect to TFA (from 18.78% to 7.27%), though it remained almost constant in EFA (17.97%). The total MUFA showed a significant decrease between TFA (52.51%) and PUFAE (36.19%), while no significant variation was observed in EFA (51.84%) (Table 6).

The parameter R, defined as the concentration of EPA plus DHA to that of 16:0 plus 18:1 [72], confirmed the increase in PUFAE from 0.74 to 2.45 in TFA, while it was constant in EFA (0.68) (*p* < 0.05) (Table 6). Similarly, the ratio between PUFA and the short-chain and saturated fatty acid percentage contents also increased in PUFAE respect to TFA (from 1.54 to 7.79) (Table 6). As a consequence, respect to TFA, the content of EPA and DHA increased (*p* < 0.05) in PUFAE from 1.58 to 2.78, respectively. The PUFA trend showed a general enrichment of 1.97 (Table 6). Breivik et al. [73], applying SPD at 125 °C on fatty acid ethyl esters from sardine oil, obtained an enrichment of 1.77 folds in EPA and of 1.6 fold in DHA. More recently, Valverde et al. [74] obtained an enrichment of 1.8 folds in EPA at 200 °C.

Our data, according to those reported for different oils [70,71,74,75,76], confirmed that SPD is an effective separation technology that can be used to concentrate PUFAs, in particular EPA and DHA, as ethyl esters from fish oil.

### 2.5. In Vitro Bioactive Properties of the Refined Oil

Many studies have underlined the beneficial effects of fish oils in cellular homeostasis, oxidative stress, and cardiovascular disease prevention and general health [77,78], thus rendering the test of these properties for new produced oils very useful in view of its commercialization. An in vitro test is an ideal and consolidated experimental model system that is able to confirm in reliable and fast way some bioactive properties exerted by natural compounds, such as refined oils, in view of its further industrial applications [35,38,79]. In view of these considerations, toxicity, antioxidant properties, and modulation of adipogenesis were the main issues we considered in our work to address these oils in human consumption and fish meal inclusion.

**Figure 3:**
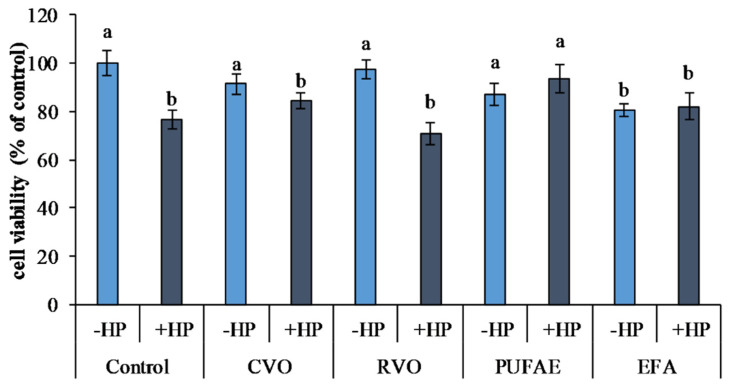
Effects of hydrogen peroxide (HP) (50 µM) induced oxidative stress and treatment with crude viscera oil (CVO), refined viscera oil (RVO), PUFA-enriched fraction (PUFAE), and short chain and unsaturated fatty acid-enriched fraction exhausted in fatty acid ethyl esters ((EFA) on the viability of 3T3 L1 cells. Different letters indicate significant differences (*p* < 0.05).

The effects of CVO, RVO, PUFAE, and EFA on the viability of 3T3 L1 cells exposed to an oxidative stress induced by hydrogen peroxide (H_2_O_2_; HP) treatment are reported in Figure 3.

Oxidative stress induced by hydrogen peroxide (control plus HP) determined a significant reduction of viability (*p* < 0.05) with respect to untreated cells (control minus HP) (Figure 3) in 3T3 L1 cells.

Compared to the controls not exposed to oxidative stress (control minus HP), treatments with CVO (CVO minus HP), RVO (RVO minus HP), and PUFAE (PUFAE minus HP) in cells not exposed to oxidative stress did not undergo a variation in cell viability. In 3T3 L1 cells exposed to EFA without oxidative stress induction (EFA minus HP), a significant reduction of viability (*p* < 0.05) was observed compared to both controls and CVO, RVO, and PUFAE treatments without oxidative stress induction (Figure 3).

Oxidative stress induction by hydrogen peroxide determined a viability reduction in control cells (control plus HP) and cells treated with CVO, RVO, and EFA (CVO plus HP, RVO plus HP, and EFA plus HP), compared to the controls not exposed to oxidative stress (control minus HP) (Figure 3).

On the contrary, the preliminary treatment of 3T3 L1 cells with PUFAE exerted a marked protective effect. Furthermore, viability was higher in PUFAE and HP with respect to the control plus HP and all other treatments exposed to oxidative stress (CVO plus HP, RVO plus HP, and EFA plus HP) (*p* < 0.05) (Figure 3).

The reported results (Figure 3) suggested that treatment with PUFA exerted a protective effect against oxidative stress, as described by Sakai et al. [36] on human aortic endothelial cells, where a significant reduction of cellular mortality after oxidative stress induced by hydrogen peroxide after EPA and DHA treatment was reported [36].

Similarly, Kusunoki et al. [35] observed a protective effect of ω-3 PUFA on 3T3-L1 against hydrogen peroxide-induced oxidative stress, confirming the well-known beneficial properties of these bioactive molecules for human health [35].

The bioactive properties of the PUFAE were also studied in fish cell line adipogenesis by evaluating morphology variation during the differentiation of *D. labrax* pre-adipocytes induced by PUFAE and EFA.

**Figure 4:**
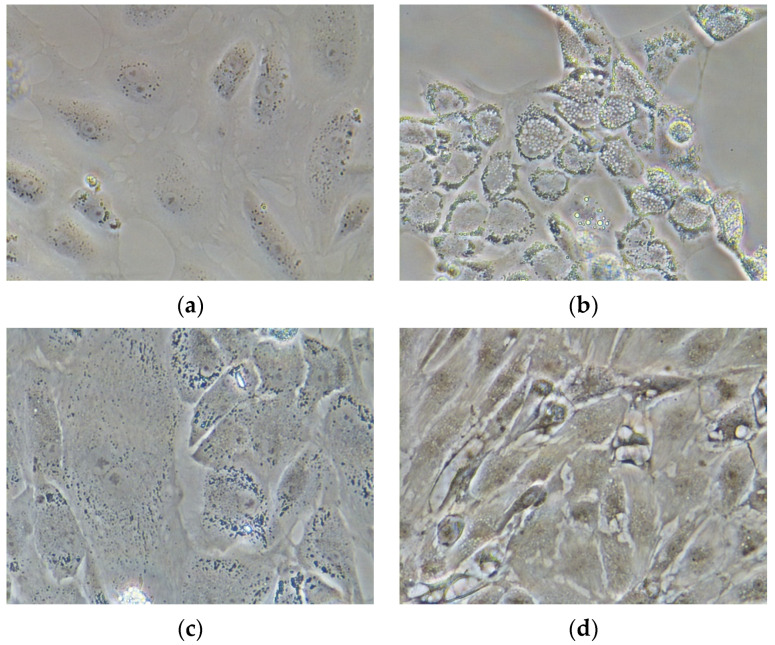
*D. labrax* pre adipocytes under differentiation (representative phase-contrast images at 40× magnification): (**a**) control undifferentiated cells; (**b**) differentiated adipocyte, induced by standard differentiation medium; (**c**) differentiated adipocyte-induced by medium supplemented with PUFAE; (**d**) differentiated adipocytes, induced by medium supplemented with EFA.

*D. labrax* pre-adipocytes started to differentiate after cell culture confluence (sixth day), in presence of factors-stimulating adipogenesis in the culture medium (L15); the differentiation was attested by the accumulation of lipid drops (Figure 4b); on the contrary, *D. labrax* pre-adipocytes, deprived of the adipogenic differentiation inducers in the culture medium, did not differentiate (Figure 4a).

In *D. labrax* pre-adipocyte culture, the presence of PUFAE, after the initial steps of differentiation, stopped the increases volume and number of lipid droplet (Figure 4c), and the resulting undifferentiated adipocytes were almost similar to the undifferentiated controls (Figure 4a) in both form and size, indicating the anti-adipogenic effect of this treatment. On the contrary, EFA treatment promoted a significant lipid accumulation (Figure 4d). The accumulation of cytoplasmatic lipid droplets was moderate during the first three days, but it increased significantly on the fourth day up to a total adipocyte hypertrophy on the seventh day (Figure 4d), thus suggesting that EFA may stimulated fat uptake and fat cytoplasm accumulation [38].

These findings were in agreement with results describing the anti-adipogenic effects of EPA and DHA during induced pre-adipocyte differentiation in cobia (*Rachycentron canadum*) [79], Atlantic salmon [80], and rainbow trout [38].

It is well-known that the fatty acid composition of the diet influences the fatty acid composition of fish [33,41,43,47], and that this well-recognized property represents the basis of the artificial diet formulations in aquaculture that are aimed to increase growth performance while maintaining high quality. Growth performance and quality are mainly influenced by the energy content of the artificial diets, integrated by the addition of vegetal oils, rich in monounsaturated and omega-6 fatty acids, by patterns of lipid distribution and metabolic management [43,45,81]. These formulations and the consequent high caloric contents are often responsible of the excessive fat deposition in farmed fish compared to the wild [43,45], which is recognized as the principle issue related to oxidative stress and consequent peroxidation due to its negative effects on fish welfare, quality, and consumer perception. In this sense, research actions aimed to preliminarily evaluate the effects of oil composition on antioxidant prevention and lipid deposition are useful to assess the nutritional properties of new formulations.

Our in vitro observations confirmed that PUFAs have a significant effects on lipid metabolism and can influence the deposition of lipids in adipocytes, suggesting a possible effect on fat deposition in fish fillet. Therefore, regulating the composition of fatty acids in the diet could strategically change the lipid deposition in various tissues and, consequently, the lipid profile of the edible parts of fish.

However, further studies are needed to understand how, through dietary manipulation, it is possible to modulate the adiposity in fish fillets in order to obtain a higher quality product.

## 3. Materials and Methods

### 3.1. Sampling

**Figure 5:**
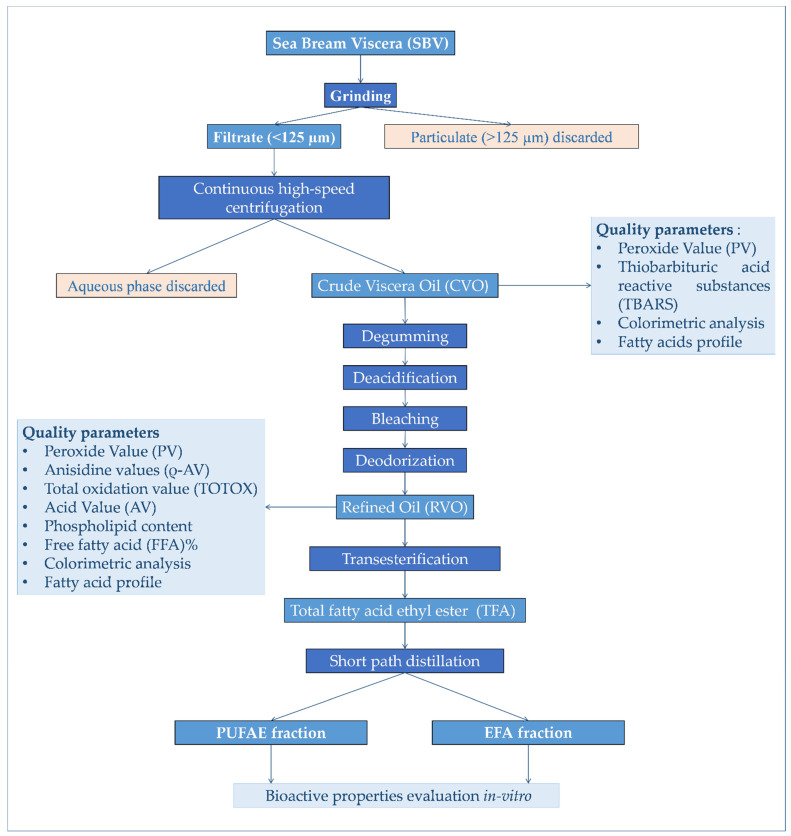
Experimental design adopted for the processing and production of ω-3-enriched-oil, as well as the evaluation of its bioactivity in vitro.

SBV was sampled at the “Acqua Azzurra s.r.l”. intensive aquaculture and fish processing farm (Pachino, SR, Italy), immediately placed on ice, transported to the laboratory, grinded, divided into aliquots of 500 g, and stored in zip-lock polyethylene bags at −80 °C, pending further analysis; treatments as summarized in Figure 5.

### 3.2. Proximate Composition and Fatty Acid Profile

The SBV proximate composition was evaluated by determining water and ash [82], crude protein [83], and total lipid [84] contents.

The fatty acid methyl ester profile of viscera lipids was determined, after transesterification, by GC using a Perkin Elmer Clarus ^®^ 580 gas chromatograph (Perkin Elmer, Shelton, CT, USA) under previous conditions [21].

### 3.3. Extraction of Crude Oil

CVO was extracted from 20 kg SBV batches by wet extraction [49].

Preheated distilled water was added to ground SBV at a 1:2 *w*/*v* ratio, and the mixture was incubated in a 50 L steel reactor equipped with an internal heating coil.

Extraction trials were performed under constant agitation at different temperatures (40, 60, 80, and 90 °C) and for different reaction periods (10, 30, and 60 min).

Extraction mixtures were filtered on a 125 µm mesh sieve to remove the coarse particulates. The filtrate liquid phase was centrifuged at a centrifugal force of 40,000× *g* by a continuous tubular centrifuge (CEPA, Carl Padberg, Zentrifugenbau GmbH, Lahr/Schwarzwald, Germany) equipped with a separating cylinder (type TR).

The extraction mixture was fed at the bottom of the cylinder by a Masterflex L/S peristaltic pump equipped with tubing L/S 18 (Cole-Parmer s.r.l., Mi, Italy), with a throughputs of 0.03 L min^−1^. This configuration allowed us to separate contemporary and continuously solids (retained in the cylinder), a heavy liquid phase (containing protein and cellular end tissue debris), and a light liquid phase (containing CVO) that exited the cylinder in two separate fluxes. CVO samples were stored at −20 °C in 2.5 L dark bottles under nitrogen.

### 3.4. Chemical Refining Process of CVO

The CVO refining process (Figure 5) was carried out in 5 L batches. During the refining process, when required, all liquid phase separation was carried out by a continuous tubular centrifuge equipped with a separating cylinder, as described above.

CVO was degummed and neutralized following the procedure of Chakraborty and Joseph [61].

After degumming and neutralization, CVO bleaching was done to remove color compounds by treatment through a column of activated charcoal powder (5% *w*/*w* of CVO).

Bleached CVO was deodorized at low temperatures by a VLK 70-4 short path distillation unit (VTA Gmbh; Niederwinkling, Germany). Bleached CVO was kept at 60 °C during the process and continuously fed into the feed vessel by a peristaltic pump (feeding rate 3 L/h) at an evaporator temperature of 120 °C, a condenser temperature of 25 °C, a vacuum of 5 mbar, and a rotor speed of 400 rpm.

At the end of the refining process, RVO was collected as the residual phase of SPD and stored at −20 °C in 2.5 L dark bottles under nitrogen, pending further analyses and processing.

### 3.5. Assessment of Oil Quality

The refining process was evaluated using commercial cod liver oil as the CO (Pearson, Campo Ligure, Ge, Italy). The quality of CVO and RVO was evaluated during the various refining steps by monitoring PV, TBARS, ρ-AV, TOTOX, phospholipid content, and free fatty acid (FFA)%, as well as through colorimetric analysis.

#### 3.5.1. Peroxide Value (PV)

Aliquots (1.0 g) of oil samples were used for PV determination. PV, expressed in milliequivalents of active oxygen per kg of oil (meqO_2_/kg), was evaluated by iodometric titration with a standard solution of sodium thiosulphate [85].

#### 3.5.2. Thiobarbituric Acid Reactive Substances Analysis (TBARS)

TBARS analysis was carried out on 0.1 g aliquots of oil following the work of Botsoglou et al. [86]. Spectrophotometric quantification was performed at 532 nm with a UV–Vis spectrophotometer (Cary 50, Varian Inc., Palo Alto, CA, USA) using a standard curve of MDA (0.001–0.5 µg/mL 5% trichloroacetic acid), and the results are expressed as content of MDA µg/g.

#### 3.5.3. Content of ρ-Anisidine (ρ-AV)

The ρ-AV was assessed according to the official AOCS method [87] slightly modified by Honold et al. [48]. Spectrophotometric quantification was performed by assessing the absorbance of each sample (0.5 g) at 350 nm (As) using a UV–Vis spectrophotometer (Cary 50, Varian Inc., Palo Alto, CA, USA) against a blank (Ab) (5 mL Chloroform with 1 mL ρ-anisidine solution) (Sigma, Aldrich).

The ρ-AV calculation is given by Equation (1):

(1) ρ−AV=(25x(1.2As−Ab))/(sample weight (g))

#### 3.5.4. Total Oxidation Value (TOTOX)

TOTOX was determined according to Holm [88] and calculated using Equation (2):

(2) TOTOX=(2×PV)+ρ−AV

#### 3.5.5. Phospholipid Content

The phospholipids, in crude and refined oil, were separated from the total triglycerides by siliceous matrix columns (HF Bond Elut SI, 100 mg, 1 mL, Varian, Palo Alto, CA, USA) by adding 0.1 g of CVO and RVO diluted in 1 mL N-hexane to the top of column and flushing the column with 20 mL of chloroform to separate triglycerides in the eluate. The column was then flushed with 20 mL of methanol and the eluate to recover phospholipids, the contents of which were then determined gravimetrically.

#### 3.5.6. Acid Value

Acid value was evaluated on aliquots of 1 g of oil sample according to the official AOCS method [89] by acid–base titration using an ethanol solution of potassium hydroxide (KOH 0.1 N in ethanol 96%) as the titrant and phenolphthalein as the indicator.

The acid value was expressed as % oleic acid equivalent according to Formula (3) [90]:

(3) %FFA (oleic acid)=((Vc−Vb)×N×28.8)/(sample weight (g))

where Vc is volume of the titrant solution used for the sample, Vb is the volume of the titrant solution for the blank, and N is KOH concentration.

#### 3.5.7. Colorimetric Analysis

The color analysis was performed following the work of Sathivel et al. [49] on three replicates of each sample (1 mL) by a Konika Minolta CR 400 spectrophotometer (Konica Minolta Chroma Co., Osaka, Japan). Results are expressed by the parameters of L*, a*, and b* and by derived variables of color saturation (C*), hue angle (h), and total color variation (ΔE) [55].

### 3.6. PUFA Enrichment

Batches of 2.5 L of RVO were trans esterified to obtain ethyl esters TFAs without the use of any solvent other than ethanol [91].

During transesterification, all liquid phase separations were carried out by a continuous tubular centrifuge equipped with a separating cylinder, as described above.

Batches of 2 L of TFA were distilled by SPD using the VLK 70-4 molecular distillation unit (VTA Gmbh, Niederwinkling, Germany) with an evaporating surface of 4.8 dm^2^.

Before PUFA enrichment, to remove impurities and any solvent traces, TFA underwent a degassing step.

TFA samples, preheated to 40 °C, were loaded into the feed vessel (at 40 °C) by a peristaltic pump. Distillation trials were run utilizing the following operating conditions: feeding vessel at 40 °C, condenser at 25 °C; evaporator at 140, 150, or 160 °C (120 °C for degassing); feeding rate of 300 mL/h (500 mL/h for degassing); roller speed of 400 rpm; and vacuum of <0.01 mbar (5 mbar for degassing).

PUFAE (heavy phase) and EFA (distilled phase) were collected, and yields were determined gravimetrically.

In order to evaluate the enrichment process every 1.0 L TFA feed and at the end of distillation, aliquots of the two separated phases were diluted at 1% in c-hexane to analyze fatty acid profiles by GC.

On the basis of the fatty acid profile, the following indices were calculated:

- EPA and DHA%.
- Fatty acid ratio ^®^ (4) [72]:
  (4) R=([EPA]+[DHA])/([16:0]+[18:1 ω9])
- Enrichment factor for EPA, DHA, and PUFA.
- Ratio of total PUFA to total saturated fatty acids (PUFA/saturated).

### 3.7. In Vitro Bioactive Properties

3T3 L1 mouse cell lines (ECACC n. 86052701, Sigma^®^ (Sigma-Aldrich, Saint Louis, MO, USA) were cultured in 75 cm^2^ plastic flasks (Nunc, Darmstadt, Germany) in Dulbecco’s Modified Eagle’s Medium (DMEM) supplemented with 10% calf serum, 2 mM glutamine, and 100 µg/mL of penicillin–streptomycin (all reagents from Sigma-Aldrich, Saint Louis, MO, USA); they were incubated in a humidified atmosphere (CO_2_ 5%).

Cells were seeded in 96-well plates at a concentration of 7 × 10^3^ cells/well and incubated for 24 h. After 24 h, the cells were treated (three replicates) with CVO, RVO, PUFAE, and EFA dissolved in ethanol at a concentration of 5 µg/mL in the medium, with a final solvent concentration of 0.1% (*v*/*v*), and left to incubate for 24 h.

As attested by internal routine procedures [92], ethanol did not exert any detrimental effects when used as vehicle.

After a preliminary test, aimed to assess the dose/dependent toxicity of the oils in a concentration range of 1–5 µg/mL, a final concentration of 5 µg/mL was selected for the bioactivity test.

After 24 h of incubation, control cells and cells incubated with CVO, RVO, PUFAE, and EFA were exposed to oxidative stress by 50 μM hydrogen peroxide, according to a previous standardized protocol [92,93,94,95], and incubated at 37 °C for 2 h.

The viability was determined by the [3-(4,5-dimethylthiazol-2-yl)-2,5-diphenyltetrazolium bromide] (MTT) method according to Mosman [96]. The optical densities (ODs) at 570 nm with background subtraction at 690 were determined in a microplate reader (Multiscan-Sky Microplate Reader, Thermo-Scientific TM, USA).

The percentage of viability was determined by Formula (5):

(5) $$Viability\;\left(\%\right)=\left(\frac{OD\;of\;the\;test\;sample}{OD\;of\;the\;control\;sample}\right)\times 100$$

OD measurements were performed in triplicate.

*D. labrax* pre-adipocytes were maintained in an L-15 Leibowitz medium (Sigma, London, UK) supplemented with 10% fetal bovine serum (FBS, Sigma, UK), 2 mM L-glutamine (Sigma, UK), 10 mM 4-(2-hydroxyethyl)-1-piperazine ethane sulfonic acid (HEPES; Sigma, UK), and penicillin–streptomycin solution (Sigma, UK), and they were seeded in 25 cm^2^ plastic tissue culture flasks (Nunc, Germany). The cells were kept at 20 °C, and the growth medium was changed every 2–3 days. The cell culture reached confluence after approximately 1 week.

The differentiation of *D. labrax* pre-adipocytes was induced by the slightly modified differentiation-inducing medium described by Todorčević et al. [37].

The L-15 medium was supplemented with 1 μM dexamethasone, 1 μM isobutylmethylxanthine, 20 μg/mL of insulin, and 0.2 µL/mL of lipid mixture, corresponding to 45 mg/mL of cholesterol and 100 mg/mL of cod liver oil ethyl esters instead of methyl esters [37].

To study its effect on pre-adipocyte differentiation, the confluent pre-adipocyte culture was incubated as follows: (1) L15 medium deprived of adipocyte differentiation inducers (control), (2) pre-adipocyte standard differentiation medium, (3) pre-adipocyte standard differentiation medium supplemented with 0.2 µL/mL of PUFAE instead of 0.2 µL/mL of lipid mixture, (4) pre-adipocyte standard differentiation medium supplemented with 0.2 µL/mL of EFA instead of 0.2 µL/mL of lipid mixture.

The medium was changed every three days until the cells reached the final step of differentiation (the morphology of mature adipocytes) (21 days) [37,97].

#### Image Acquisition

Cells in the culture were observed daily using a Nikon Eclipse Ti-S inverted microscope (Nikon Instrument Inc., Melville, NY, USA), and images were captured by a Nikon DS-L3 digital camera (Nikon Corporation, Tokyo, Japan) and the DS-L3 Digital Camera Controller acquisition software. Images represent pre-adipocytes cells observed with phase contrast microscopy at 40× magnification.

### 3.8. Statistical Analysis

Results are reported as mean ± standard deviation. Observed differences among the effects of temperature and extraction time during the extractions were analyzed by an ANOVA [98]. Differences observed among results of the refining process were analyzed by Student *t* tests. Cochran′s C test was used to test the assumption of variance homogeneity. Student–Newman–Keuls (SNK) post hoc tests were conducted for all significant interaction terms [98]. The differences were considered significant for *p* < 0.05. All elaborations were performed using STATISTICA 7.0 (Statsoft Inc., Tulsa, OK, USA).

## 4. Conclusions

There is a wide and extensive body of literature attesting to the presence and properties of the bioactive compounds contained in marine discard, wastes, and processed by-products and that stimulate their utilization in diverse industrial sectors; moreover, there is a discrepancy between laboratory and industrial applications, as most results are valid only at the lab scale. This situation is responsible for the delay in innovation in the sector of marine biotechnology and blue-economy, which needs the implementation of procedures and protocols with high technological readiness levels (TRLs) in order to boost productivity and competitiveness. The present paper attests to the validity of the pilot protocol for processing high volumes of industrial aquaculture by-products in high volumes of enriched fish oil in view of its application in the real world. This example could stimulate the adoption of solutions aimed to recover and utilize aquaculture by-products at a higher scale, turning “waste into profit” and indicating a strategy to reach more sustainable business models in aquaculture resource utilization according to the principles of the circular economy.

We started from an extensive study on the chemical and nutritional characterization of sea bream by-products (crude and enriched oil extracted from SBV), followed by bioactivity evaluation.

The obtained results showed that SBV is a very suitable source among sea bream waste because it is very rich in lipids and therefore an excellent matrix for the production of fish oils. Crude oils showed good characteristics, and refined oils were found to be adequate for a direct human consumption according to the current European Food Safety Authority (EFSA) guidelines. In addition, SPD was found to be a simple, economical, and environmentally sustainable technique that resulted in a product containing up to 56% long-chain PUFAs. Furthermore, the in vitro approach represents a fast and reliable way to appreciate the effects of oil in cells in view of its application for nutraceutical uses and feed formulations in aquaculture to ensure an optimal degree of reared fish adiposity.

Our study confirms the potential recycling of fish by-products for conversion into products of higher value and the reduction of the impact of the aquaculture sector, improving its economic performance and conforming to zero waste strategies.

## Figures and Tables

**Table 1:** Proximate composition of sea bream viscera (SBV) by-product (mean ± standard deviation values; *n* = 12).

Parameters	g/100 g SBV
Lipid	51.79 ± 12.92
Moisture	40.81 ± 4.86
Protein	5.67 ± 0.02
Ash	1.43 ± 0.55

**Table 2:** Fatty acid profile (%) of the total lipids in sea bream viscera (SBV) (mean ± standard deviation values; *n* = 12). EPA: eicosapentaenoic acid; DHA: docosahexaenoic acid; SFA: saturated fatty acid; MUFA: monounsaturated fatty acids; PUFA: polyunsaturated fatty acid.

Fatty Acids	%
14:0	4.99 ± 0.17
16:0	15.56 ± 0.18
16:1n−7	10.72 ± 0.18
16:2n−4	0.89 ± 0.03
16:3n−4	0.59 ± 0.02
18:0	2.96 ± 0.06
18:1n−9	16.11 ± 0.40
18:1n−7	6.05 ± 0.15
18:2n−6	2.42 ± 0.16
18:3n−4	0.01 ± 0.00
18:3n−3	0.62 ± 0.04
18:4n−3	1.68 ± 0.06
20:1n−9	11.14 ± 0.27
20:4n−6	0.13 ± 0.03
20:4n−3	0.38 ± 0.08
EPA	8.19 ± 0.12
22:1n−11	4.71 ± 0.17
22:1n−9	0.38 ± 0.02
22:5n−3	1.19 ± 0.08
DHA	11.27 ± 0.30
SFA	23.51 ± 0.28
MUFA	49.12 ± 0.76
PUFA	27.38 ± 0.48

**Table 3:** Quality of crude viscera oil (CVO) extracted at different temperatures (40, 60, 80, and 90 °C) and different extraction times (10′, 30′, and 60′), as determined by the evaluation of peroxide value (PV; meqO_2_/kg) and thiobarbituric acid reactive substances (TBARS) (MDA µg/g). Commercial cod liver oil was used as the control oil (CO).

Sample		Parameters	
Temperatures	Times	PV	TBARS
40	10	6.17 ± 0.47 ^b^	16.01 ± 0.02 ^b^
	30	6.51 ± 0.22 ^b^	15.59 ± 0.03 ^b^
	60	10.29 ± 2.37 ^c^	21.18 ± 5.85 ^bc^
60	10	5.42 ± 0.13 ^b^	14.15 ± 0.07 ^b^
	30	6.38 ± 0.03 ^b^	16.58 ± 0.01 ^b^
	60	11.33 ± 0.75 ^cd^	14.31 ± 0.22 ^b^
80	10	11.01 ± 1.77 ^cd^	17.50 ± 2.41 ^b^
	30	13.27 ± 2.13 ^d^	25.38 ± 8.59 ^c^
	60	19.69 ± 0.90 ^e^	19.89 ± 2.29 ^bc^
90	10	10.99 ± 0.53 ^cd^	31.41 ± 6.33 ^d^
	30	19.67 ± 2.10 ^e^	33.09 ± 1.31 ^d^
	60	22.74 ± 2.27 ^f^	33.43 ± 5.63 ^d^
CO		2.10 ± 0.53 ^a^	5.51 ± 0.81 ^a^

Different superscript letters in the same column indicate significant differences (a, b, c…: *p* < 0.05). The data are reported as mean ± standard deviation (*n* = 12).

**Table 4:** Main classes of fatty acids (%) extracted from sea bream viscera (SBV) and crude viscera oil (CVO) after extraction at different temperatures (°C) and reaction times (min).

Samples		SFA	MUFA	PUFA	EPA	DHA
SBV		23.51 ± 0.28 ^a^	49.12 ± 0.76 ^ab^	27.38 ± 0.48 ^e^	8.19 ± 0.12 ^eg^	11.27± 0.30 ^e^
CVO						
Temperature	Time					
40	10	24.98 ± 1.03 ^ab^	50.09 ± 0.66 ^b^	24.93 ± 0.37 ^bcd^	7.12 ± 0.52 ^bcd^	10.15 ± 0.06 ^cd^
	30	24.26± 0.58 ^a^	50.28 ± 0.15 ^b^	25.46 ± 0.58 ^cde^	7,78 ± 0.18 ^def^	10.03 ± 0.36 ^cd^
	60	26.61 ± 1.35 ^bc^	49.81 ± 0.28 ^b^	23.58 ±1.64 ^bc^	6.91 ± 0.51 ^bc^	9.25 ± 1.00 ^bc^
60	10	25.28 ± 0.01 ^ab^	49.84 ± 0.11 ^b^	24.88 ± 0.10 ^bcd^	7.54 ± 0.05 ^cdef^	9.69 ± 0.02 ^bcd^
	30	25.69 ± 1.41 ^abc^	49.43 ± 0.06 ^b^	24.88 ± 1.36 ^bcd^	7.71 ± 0.56 ^def^	9.61 ± 0.49 ^bcd^
	60	25.17 ± 0.79 ^ab^	48.86 ± 0.49 ^b^	25.97 ± 1.28 ^de^	7.92 ± 0.69 ^ef^	9.85 ± 0.67 ^cd^
80	10	26.62 ± 1.66 ^bc^	47.41± 0.69 ^a^	25.97 ± 2.35 ^de^	7.56 ± 0.35 ^cdef^	10.49 ± 1.37 ^de^
	30	27.23 ± 0.80 ^c^	49.83 ± 0.42 ^b^	22.94 ± 0.37 ^b^	6.73 ± 0.08 ^b^	8.71 ± 0.37 ^ab^
	60	24.78 ± 0.77 ^a^	49.42 ± 0.38 ^b^	25.79 ± 1.15 ^cde^	7.38 ± 0.56 ^bcde^	10.61 ± 0.54 ^de^
90	10	24.48 ± 0.62 ^a^	48.54 ± 0.33 ^ab^	26.98 ± 0.97 ^de^	8.64 ± 0.42 ^g^	10.40 ± 0.52 ^cde^
	30	25.25 ± 0.76 ^ab^	48.72 ± 0.39 ^b^	25.76 ± 0.84 ^cde^	7.44 ± 0.30 ^cdef^	9.68 ± 0.45 ^bcd^
	60	26.69 ± 0.21 ^bc^	52.30 ± 2.75 ^c^	21.02 ± 1.90 ^a^	6.09 ± 0.45 ^a^	7.97 ± 0.43 ^a^

Different superscript letters in the same column indicate significant differences (a, b, c…: *p* < 0.05). The data are reported as mean ± standard deviation (*n* = 12).

**Table 5:** Effect of crude viscera oil (CVO) extraction temperature (60 and 80 °C) on the quality of refined viscera oil (RVO), as determined by peroxide value: PV; ρ-anisidine: ρ-AV; thiobarbituric acid reactive substances: TBARS; total oxidation value: TOTOX; phospholipids; % free fatty acid: %FFA. Commercial cod liver oil was used as the control oil (CO).

		Parameters					
Samples	CVO Extraction Temperature	PV (meqO_2_/kg)	ρ-AV	TBARS (MDA µg/g)	TOTOX	Phospholipids (mg kg^−1^)	Acid Value (%FFA)
CVO	60 °C	5.41 ± 0.14 ^b^	28.98 ± 0.90 ^d^	14.15 ± 0.07 ^d^	39.80 ± 1.18 ^c^	24.75 ± 1.61 ^d^	8.23 ± 0.82 ^d^
RVO		3.90 ± 1.15 ^b^	13.49 ± 0.20 ^b^	6.62 ± 0.39 ^b^	21.30 ± 2.50 ^b^	8.00 ± 1.10 ^a^	1.74 ± 0.33 ^b^
CVO	80 °C	11.17 ± 1.31 ^d^	34.56 ± 2.36 ^e^	17.50 ± 2.41 ^e^	56.90 ± 0.26 ^e^	47.47 ± 14.05 ^e^	7.41 ± 0.49 ^c^
RVO		8.63 ± 0.31 ^c^	24.06 ± 1.13 ^c^	8.96 ± 0.86 ^c^	41.32 ± 0.51 ^d^	14.42 ± 3.90 ^c^	6.62 ± 0.74 ^c^
CO		2.10 ± 0.53 ^a^	4.96 ± 0.89 ^a^	5.51 ± 0.81 ^a^	10.83 ± 0.24 ^a^	10.74 ± 2.45 ^b^	0.50 ± 0.04 ^a^

Different superscript letters in the same column indicate significant differences (a, b, c…: *p* < 0.05). The data are reported as mean ± standard deviation (*n* = 12).

**Table 6:** Fatty acid profile (% fatty acids) and enrichment indexes of the ethyl esters of the total fatty acid (TFA), the fraction enriched in PUFA (PUFAE), and the fraction exhausted in fatty acid ethyl esters (EFA), as obtained by short path distillation (SPD) at 160 °C. The data are reported as mean ± standard deviation.

Fatty Acids	TFA	PUFAE	EFA
14:0	5.07 ± 0.57 ^b^	0.66 ± 0.28 ^a^	4.35 ± 0.05 ^b^
16:0	11.47 ± 0.96 ^b^	4.45 ± 0.46 ^a^	11.26 ± 0.03 ^b^
16:1n−7	8.63 ± 2.10 ^b^	2.43 ± 0.16 ^a^	7.48 ± 0.02 ^b^
16:2n−4	0.86 ± 0.02 ^b^	0.38 ± 0.21 ^a^	1.01 ± 0.06 ^b^
16:3n−4	0.48 ± 0.06 ^b^	0.22 ± 0.12 ^a^	0.65 ± 0.08 ^b^
18:0	2.24 ± 0.19	2.15 ± 0.28	2.36 ± 0.23
18:1n−9	16.58 ± 0.87 ^b^	14.72 ± 0.77 ^a^	18.32 ± 0.27 ^b^
18:1n−7	5.71 ± 1.07	3.90 ± 0.66	5.27 ± 0.57
18:2n−6	1.48 ± 0.13	1.17 ± 0.75	1.78 ± 0.06
18:3n−4	0.01 ± 0.01 ^a^	0.02 ± 0.01 ^a^	0.17 ± 0.00 ^b^
18:3n−3	0.69 ± 0.11 ^a^	0.60 ± 0.19 ^a^	0.95 ± 0.02 ^b^
18:4n−3	2.23 ± 0.11 ^b^	1.61 ± 0.10 ^a^	2.48 ± 0.14 ^c^
20:1n−9	12.67 ± 0.83 ^b^	5.51 ± 0.95 ^a^	11.44 ± 0.05 ^b^
20:3n−3	0.22 ± 0.19	0.48 ± 0.18	0.43 ± 0.00
20:4n−6	0.16 ± 0.08	0.20 ± 0.14	0.26 ± 0.00
20:4n−3	0.53 ± 0.11 ^a^	1.09 ± 0.25 ^b^	0.97 ± 0.01 ^b^
EPA-20:5n−3	8.80 ± 0.63 ^a^	13.92 ± 1.05 ^b^	9.28 ± 0.03 ^a^
22:1n−11	8.33 ± 1.95 ^b^	6.67 ± 0.28 ^a^	8.31 ± 0.19 ^b^
22:1n−9	0.59 ± 0.39 ^a^	2.96 ± 0.38 ^b^	1.03 ± 0.05 ^a^
22:5n−3	1.39 ± 0.11 ^a^	3.97 ± 0.42 ^b^	1.40 ± 0.05 ^a^
DHA-22:6n−3	11.84 ± 0.78 ^a^	32.90 ± 0.76 ^b^	10.78 ± 0.19 ^a^
SFA	18.78 ± 1.29 ^a^	7.27 ± 0.17 ^b^	17.97 ± 0.19 ^a^
MUFA	52.51 ± 0.95 ^b^	36.19 ± 0.88 ^a^	51.84 ± 0.24 ^b^
PUFA	28.71 ± 2.11 ^a^	56.55 ± 0.93 ^b^	30.19 ± 0.14 ^a^
R	0.74 ± 0.05 ^a^	2.45 ± 0.20 ^b^	0.68 ± 0.01 ^a^
EPA Enrichment Factor	1.00 ± 0.07 ^a^	1.58 ± 0.12 ^b^	1.05 ± 0.00 ^a^
DHA Enrichment Factor	1.00 ± 0.07 ^a^	2.78 ± 0.06 ^b^	0.91 ± 0.02 ^a^
PUFA enrichment facto	1.00 ± 0.07 ^a^	1.97 ± 0.03 ^b^	1.05 ± 0.00 ^a^
PUFA/SFA	1.54 ± 0.21 ^a^	7.79 ± 0.26 ^b^	1.68 ± 0.02 ^a^

Different superscript letters in the same row indicate significant differences (a, b, c…: *p* < 0.05). The data are reported as mean ± standard deviation, *n* = 12.

## Supplementary Materials

The following are available online at https://www.mdpi.com/1660-3397/19/3/160/s1, Table S1: CIELAB colour coordinates (L*, a*, b*, Chromaticity—C*, tint angle—h and total color variation—ΔE) in crude viscera oil (CVO) extracted from sea bream viscera (SBV) at different temperatures (°C) and reaction time (min). Commercial Cod liver oil (CO) was used as control. Table S2: CIELAB colour coordinates (L*, a*, b*, Chromaticity—C*, tint angle—h and total color variation—ΔE) in crude viscera oil (CVO) and refined viscera oil (RVO). Commercial Cod liver oil (CO) was used as control. Figure S1: Crude viscera oils (CVO) extracted from sea bream viscera (SBV) at different temperatures. (40 °C, 60 °C and 80 °C).
