# Supplementary material for: Farmed Gilthead Sea Bream (Sparus aurata) by-Products Valorization: Viscera Oil ω-3 Enrichment by Short-Path Distillation and In Vitro Bioactivity Evaluation
Source: Mar Drugs. 2021 Mar 18;19(3):160. doi: 10.3390/md19030160 (PMC8002999; doi:10.3390/md19030160)
Supplement: Supplementary file 1 [file marinedrugs-19-00160-s001.pdf]

marine drugs

MDPI

Supplementary materials

Table S1. CIELAB colour coordinates (L*, a*, b*, Chromaticity - C*, tint angle - h and total color variation – ΔE) in crude viscera oil (CVO) extracted from sea bream viscera (SBV) at different temperatures (°C) and reaction time (min). Commercial Cod liver oil (CO) was used as control.

**Sample**		**Parameters**				
**Temperature**	**Times**					
		*L**	*a**	*b**	hue angle (*h*)	chroma (*C**)
40	10	70.12 ± 0.04^{g}	-3.49 ± 0.03^{e}	15.94 ± 0.06^{c}	102.35 ± 0.08^{g}	16.32 ± 0.06^{c}
	30	70.54 ± 0.01^{h}	-3.93 ± 0.03^{d}	14.27 ± 0.01^{b}	105.41 ± 0.09^{n}	14.80 ± 0.01^{b}
	60	69.96 ± 0.01^{g}	-4.14 ± 0.01^{c}	17.41 ± ‘0.02^{e}	103.36 ± 0.04^{h}	17.90 ± 0.01^{e}
60	10	69.27 ± 0.01^{f}	-4.10 ± 0.03^{c}	16.89 ± 0.03^{d}	103.64 ± 0.06^{i}	17.38 ± 0.03^{d}
	30	70.81 ± 0.05^{h}	-4.60 ± 0.03^{b}	16.88 ± 0.04^{d}	105.26 ± 0.09^{m}	17.50 ± 0.04^{d}
	60	70.61 ± 0.09^{h}	-5.06 ± 0.01^{a}	19.04 ± 0.01^{f}	104.88 ± 0.03^{l}	19.70 ± 0.01^{f}
80	10	65.42 ± 0.62^{d}	-1.99 ± 0.09^{g}	35.73 ± 0.56^{h}	93.19 ± 0.10^{e}	35.79 ± 0.56^{h}
	30	66.81 ± 0.05^{e}	-3.35 ± 0.33^{f}	32.09 ± 0.05^{g}	95.95 ± 0.06^{f}	32.26 ± 0.05^{g}
	60	61.26 ± 0.01^{a}	2.25 ± 0.01^{m}	46.24 ± 0.03^{n}	87.21 ± 0.01^{a}	46.29 ± 0.02^{n}
90	10	64.48 ± 0.01^{c}	-0.72 ± 0.01^{h}	38.09 ± 0.02^{i}	91.08 ± 0.02^{d}	38.10 ± 0.02^{i}
	30	64.29 ± 0.02^{bc}	0.04 ± 0.01^{l}	44.45 ± 0.01^{l}	89.95 ± 0.01^{b}	44.45 ± 0.01^{l}
	60	64.08 ± 0.05^{b}	-0.58 ± 0.02^{i}	45.39 ± 0.02^{m}	90.74 ± 0.03^{c}	45.39 ± 0.02^{m}
CO		87.86 ± 0.19^{i}	-3.32 ± 0.01^{f}	9.81 ± 0.01^{a}	108.69 ± 0.08^{o}	10.36 ± 0.01^{a}

Different superscript letters in the same column indicate significant differences (a, b, c…: $P < 0.05$). The data are reported as mean ± standard deviation (n=12).

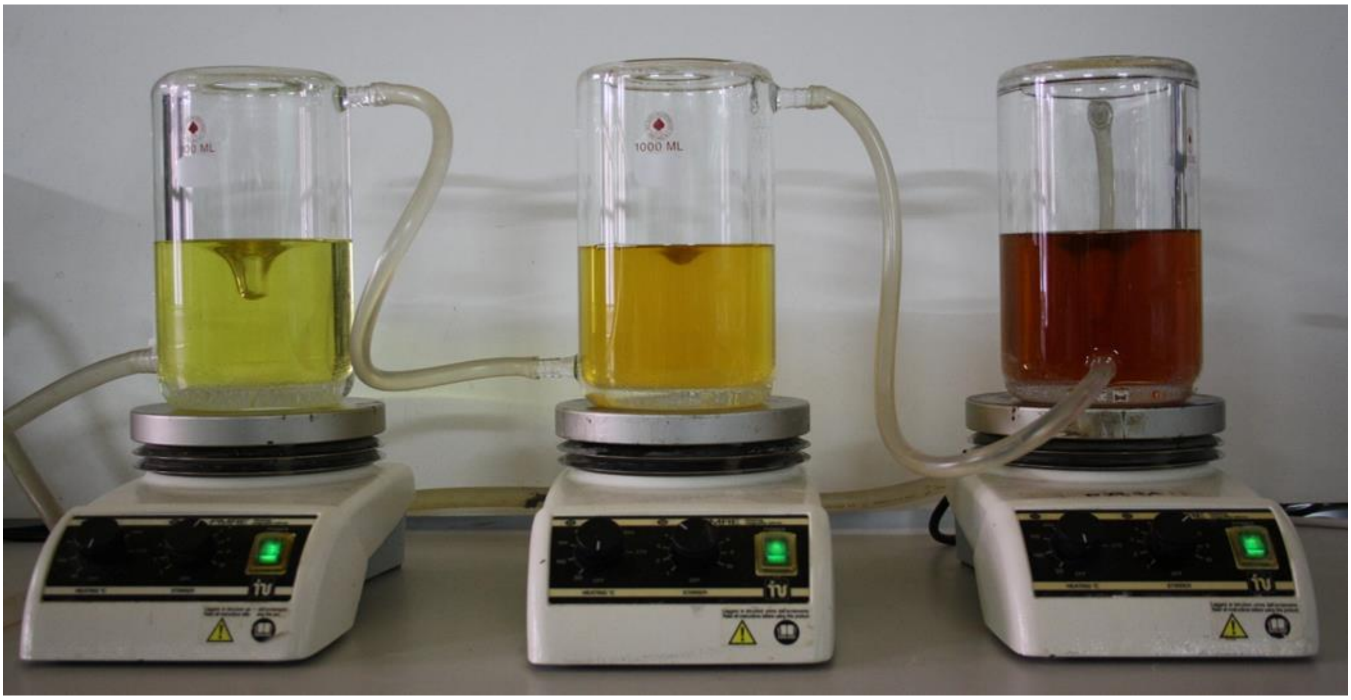

Figura S1. Crude viscera oils (CVO) extracted from sea bream viscera (SBV) at different temperatures. (40°C, 60°C and 80°C)

Table S2. CIELAB colour coordinates (L*, a*, b*, Chromaticity - C*, tint angle - h and total color variation – ΔE) in crude viscera oil (CVO) and refined viscera oil (RVO). Commercial Cod liver oil (CO) was used as control

**Sample**	**Parameters**				
	L*	***a****	***b****	hue angle (*h*)	chroma (*C**)
CVO 60°C	85.50±0.13^{c}	-4.15±0.01^{a}	18.83±0.03^{c}	102.44±0.05^{d}	19.28±0.03^{c}
RVO 60°C	86.68±0.19^{d}	-3.68±0.02^{b}	17.64±0.03^{b}	101.79±0.04^{c}	18.02±0.03^{b}
CVO 80°C	80.83±0.01^{a}	-1.10±0.02^{d}	38.75±0.02^{e}	91.63±0.02^{a}	38.76±0.02^{e}

RVO 80°C	82.48±0.94^{b}	-2.49±0.06^{c}	32.91±0.68^{d}	94.33±0.05^{b}	33.01±0.68^{d}
**CO**	87.86±0.19^{e}	3.26±0.03^{e}	10.49±0.02^{a}	107.25±0.15^{e}	10.98±0.03^{a}

Different superscript letters in the same column indicate significant differences (a, b, c…: P < 0.05). The data are reported as mean ± standard deviation (n=12).
